# *Toxoplasma gondii* Protein Disulfide Isomerase (TgPDI) Is a Novel Vaccine Candidate against Toxoplasmosis

**DOI:** 10.1371/journal.pone.0070884

**Published:** 2013-08-15

**Authors:** Hai-Long Wang, Ya-Qing Li, Li-Tian Yin, Xiao-Li Meng, Min Guo, Jian-Hong Zhang, Hong-Li Liu, Juan-Juan Liu, Guo-Rong Yin

**Affiliations:** 1 Research Institute of Medical Parasitology, Shanxi Medical University, Taiyuan, Shanxi, PR China; 2 Department of Physiology, Key Laboratory of Cellular Physiology Co-constructed by Province and Ministry of Education, Shanxi Medical University, Taiyuan, Shanxi, PR China; 3 Center of Laboratory Animal, Shanxi Medical University, Taiyuan, Shanxi, PR China; Charité, Campus Benjamin Franklin, Germany

## Abstract

*Toxoplasma gondii* is a ubiquitous protozoan parasite that can infect all warm-blooded animals, including both mammals and birds. Protein disulfide isomerase (PDI) localises to the surface of *T. gondii* tachyzoites and modulates the interactions between parasite and host cells. In this study, the protective efficacy of recombinant *T. gondii* PDI (rTgPDI) as a vaccine candidate against *T. gondii* infection in BALB/c mice was evaluated. rTgPDI was expressed and purified from *Escherichia coli*. Five groups of animals (10 animals/group) were immunised with 10, 20, 30, 40 μg of rTgPDI per mouse or with PBS as a control group. All immunisations were performed via the nasal route at 1, 14 and 21 days. Two weeks after the last immunisation, the immune responses were evaluated by lymphoproliferative assays and by cytokine and antibody measurements. The immunised mice were challenged with tachyzoites of the virulent *T. gondii* RH strain on the 14th day after the last immunisation. Following the challenge, the tachyzoite loads in tissues were assessed, and animal survival time was recorded. Our results showed that the group immunised with 30 μg rTgPDI showed significantly higher levels of specific antibodies against the recombinant protein, a strong lymphoproliferative response and significantly higher levels of IgG2a, IFN-gamma (IFN-γ), IL-2 and IL-4 production compared with other doses and control groups. While no changes in IL-10 levels were detected. After being challenged with *T. gondii* tachyzoites, the numbers of tachyzoites in brain and liver tissues from the rTgPDI group were significantly reduced compared with those of the control group, and the survival time of the mice in the rTgPDI group was longer than that of mice in the control group. Our results showed that immunisation with rTgPDI elicited a protective immune reaction and suggested that rTgPDI might represent a promising vaccine candidate for combating toxoplasmosis.

## Introduction

*Toxoplasma gondii* is a ubiquitous protozoan parasite that can infect virtually all warm-blooded animals, including both mammals and birds. Epidemiological surveys have suggested that *T. gondii* infection has a wide distribution and a high prevalence in many areas of the world. Up to one-third of the world's population is infected with this parasite [1], [2]. Ingesting uncooked meat containing tissue cysts or food and water contaminated with oocysts from infected cat faeces, and transmission of tachyzoites to foetuses through placenta are the three primary routes of transmission of *T. gondii*. Most people infected with *T. gondii* shows no symptom, but severe complications occur in pregnant women and immunocompromised individuals, such as recipient of organ transplant, AIDS patients and cancer patients undergoing chemotherapy [3].

In addition, infection of *T. gondii* in domestic animals represents a considerable threat to public health due to food-borne outbreaks leading to heavy economic loss worldwide [4]. Treatment of toxoplasmosis is difficult due to the severe side effects of the available drugs and re-infection may occur at any time [5], [6]. Therefore, development of an effective vaccine against *T. gondii* or new anti-*Toxoplasma* drugs are of great importance in preventing both foetal infection and infection of immunocompromised patients, as well as reduce the economic loss due to abortion of farm animals [7].

In recent years, extensive efforts have been made to develop anti-*Toxoplasma* vaccine, including vaccines of inactivated, attenuated, subunit, genetically engineered and DNA vaccines [8]. However, little protection has been offered against this ubiquitous pathogen. At present, only an attenuated vaccine (Toxovax, Intervet Shering-Plough) based on the live attenuated S48 strain has been licensed for use in sheep in Europe and New Zealand for over two decades [9]. The drawbacks of this vaccine are short shelf-life, adverse effects, and the potential risk of reverting to a pathogenic strain. This vaccine is apparently not suitable for human [7]. Therefore, it is important to search for novel target antigens in order to stimulate potent immunoprotection via an optimal delivery strategy.

By using bioinformatics, genomics and proteomics strategies, more novel and effective vaccine candidates have been identified [10]. *T. gondii* protein disulfide isomerase (PDI) is one of the new candidate antigens identified among soluble tachyzoite antigens using rabbit anti-*T. gondii* serum by two-dimensional gel electrophoresis and proteomics analyses [11]. PDIs not only highly expressed in *T. gondii* and *Neospora caninum* [12], [13], but are also found in virtually all eukaryotic cells in several protozoan parasites, such as *Cryptosporidium parvum* [14], *Plasmodium falciparum* [15], *Leishmania major* [16], and *Leishmania donovani* [17]. PDIs exhibit oxidoreductase, isomerase, and chaperone activities, ensuring the proper folding and conformation of the proteins by catalysing the formation or oxidation of disulfide bridges [18], [19]. PDIs are localised mainly in endoplasmic reticulum (ER) and are involved in cell signalling and homeostasis [20]. Recently, some evidences have suggested that PDIs are also present at cell surface. They functionality modulate cellular interactions with a number of pathogens [21]. For instance, PDIs from *T. gondii* and *N. caninum* have been identified at the surface of tachyzoites, and they modulate the tachyzoite-host cell interaction [22], [23]. Interestingly, 81% of individuals display an anti-*T. gondii* IgA antibody in their tears that can recognise *T. gondii* PDI (TgPDI) [24]. The antibody might be upregulated as a continuous mucosal defence against protozoan parasites [25]. These observations strongly suggest that TgPDI could serve as a candidate vaccine antigen.

To evaluate the immune responses and protective efficacy of the TgPDI as a mucosal vaccine against homologous challenge in BALB/c mice, a purified recombinant TgPDI protein (rTgPDI) expressed in *Escherichia coli* (*E. coli*) was used by us for immunisation of BALB/c mice. Specific antibodies and cytokine levels and lymphocyte proliferation were examined in these immunised mice. After the challenge with tachyzoites from the RH strain of *T. gondii*, the numbers of brain and liver tachyzoites in the BALB/c mice were recorded, and the survival time of rTgPDI-immunised mice was observed.

## Materials and Methods

### Animals and parasites

The inbred-strain mice used in this study were 6-week-old BALB/c mice purchased from the Institute of Laboratory Animals, Chinese Academy of Medical Science, Beijing. *T. gondii* tachyzoites (RH strain) were kindly provided by Peking University Health Science Center and were maintained via serial intraperitoneal passaging in BALB/c mice.

### Ethics Statement

This study was carried out in strict accordance with the recommendations of the Guide for the Care and Use of Laboratory Animals of Shanxi Medical University. Mice were bred and maintained under conventional, non-SPF conditions at the Center of Laboratory Animals with a 12-hour light/dark cycle. Water and food were provided *ad libitum*. All experimental procedures were approved by the Laboratory Animal Use and Care Committee of Shanxi Medical University (Permit Number: SXMU-2009-16) and by the Ethics Committee of Animal Experiments of Shanxi Medical University (Permit Number: 20110307-1). All surgery was performed under sodium pentobarbital anaesthesia, and all possible efforts were made to minimise suffering of experiment mice.

### Alignment of the amino acid sequence of TgPDI

The amino acid sequence of PDI from three strains of *T. gondii* was obtained from internet (http://www.ncbi.nlm.nih.gov). The protein accession number is CAC28361 for RH strain, type I; XP_002371293 for ME 49 strain, type II; EEE29915 for VEG strain, type III respectively. The alignment was performed by DNAMAN software (Lynnon, Quebec, Canada).

### Recombinant protein expression in *E. coli* and purification

Total RNA was extracted from 5×10^8^ tachyzoites according to the instructions of the manufacturer of the TRIzol reagent (CoWin Biotechnology, Beijing, CN). First-strand cDNA was synthesised using the HiFi-MMLV cDNA Kit (CoWin Biotechnology), and the coding region of TgPDI was amplified via reverse transcription polymerase chain reaction (RT-PCR). Primers for amplification of the ORF of the TgPDI gene were designed considering the TgPDI sequence of the RH strain of *T. gondii* (GenBank accession No: AJ306291.2). The forward primer was 5′-ACGCGGATCCATGCGAGCCGGGTTTTCGTTTG-3′, and the reverse primer was 5′-ACCGCTCGAGCAGTTCTTCACCCTTGTCGTCC-3′, which contained *Bam*H I and *Xho* I restriction sites (underlined), respectively. PCR was carried out according to a conventional protocol.

For recombinant protein expression in *E. coli*, the TgPDI cDNA fragment was cloned into the pET-30a(+) vector (Novagen, USA) to form pET-30a(+)-TgPDI. This pET-30a(+)- TgPDI plasmid was then transformed into Rosetta (DE3) host bacteria cells (Transgen, CN), and recombinant protein expression was induced with 0.1 mM IPTG under continuous culture at 25°C for 8 h.

The recombinant cells were harvested via centrifugation, and the obtained pellets were resuspended in lysis buffer (50 mM Tris pH 7.7, 500 mM NaCl, 10 mM imidazole, 1 mM PMSF, 2 mM DTT) and homogenised via sonication on ice. The lysate was then centrifuged to separate the supernatant and cell debris. The level of rTgPDI expression was evaluated through 12% sodium dodecyl sulphate polyacrylamide gel electrophoresis (SDS-PAGE) with Coomassie blue R-250 staining. A western blotting assay with a His primary antibody, rabbit anti-*T. gondii* serum or human anti-*T. gondii* serum (which was provided by professor Suo Xun, China Agricultural University) was performed to confirm the expression of rTgPDI. Moreover, native TgPDI from soluble tachyzoite antigen (STAg) was detected by Western blotting using the mouse anti-rTgPDI serum which was generated in this study.

All of the subsequent purification steps were performed at 4°C. The clear supernatants were mixed with Ni^2+^-NTA agarose (Qiagen, Germany) and incubated for 1 h with gentle shaking. The resultant slurry was transferred to a chromatography column, washed with 10 bed volumes of wash buffer (50 mM Tris pH 7.7, 500 mM NaCl, 20 mM imidazole), and the target protein was eluted with elution buffer (50 mM Tris pH 7.7, 500 mM NaCl, 150 mM imidazole), then pooled and transferred to desalting buffer (25 mM Tris pH 8.5, 80 mM NaCl, 2 mM DTT) using a Gravity DeSalting Column (Shanghai Sangon, CN). The desalted samples were concentrated to 5 mg/ml in sterile PBS using a 300,000 molecular weight device (Sartorius Stedim Biotech), and the purified protein was analysed via SDS-PAGE.

### rTgPDI immunisation

The immunisation experiments were performed in 6-week-old BALB/c mice, half of which were male, and half were female. Five groups of mice (10 per group) were intranasally immunised with 10, 20, 30 or 40 μg of rTgPDI dissolved in 20 μl of sterile PBS, or with PBS as a control. Each dose of immunogen (10 μl/nostril) was introduced into the nostrils of mice with a micropipette within a period of 5 minutes to reduce the distress of the mice. The mice were immunised using the same protocol on days 0, 14, and 21. Two weeks after the final inoculation (on day 35), the mice were anesthetised with sodium pentobarbital (1.5%, 0.1 ml/20 g weight). Blood samples were collected from the mice in each group through retro-orbital plexus puncture, and the sera were stored at −20°C until being used for analysis. Spleens were collected under aseptic conditions to perform lymphocyte proliferation assays, and the culture supernatants were used for cytokine assays.

### IgG and IgA assays

Enzyme-linked immunosorbent assays (ELISAs) were performed for the detection of antigen-specific IgA, IgG, IgG1 and IgG2a antibodies in serum samples collected two weeks after the last immunisation. Briefly, 96-well flat-bottom microtiter plates were coated with 1 μg rTgPDI in 100 μl sodium carbonate buffer (pH 9.2) overnight at 4°C. The plates were then washed with PBS containing 0.05% Tween 20 (PBST), pH 7.4. PBS containing 5% FCS was used for blocking non-specific binding sites for 2 h at 37°C. After washing three times (each time for 5 min) with PBST, individual sera (100 μl/well) diluted in 1% BSA-PBST (1∶150, 1∶50 for IgA, IgG1 and IgG2a) were transferred to the wells, followed by incubation for 1 h at 37°C. After the plates were washed, bound antibodies were detected by adding 50 μl of horseradish peroxidase-conjugated goat anti-mouse IgA, IgG, IgG1 or IgG2a (ProteinTech Group, Inc, USA) diluted 1∶2000. After the plates were washed in PBST, immune complexes were revealed by incubation with orthophenylene diamine (Sigma) and 0.15% H_2_O_2_ for 30 min, and the enzyme reaction was then terminated by adding 1 M H_2_SO_4_. The optical density (OD) was measured in an ELISA reader (Epoch Multi-Volume Spectrophotometer System, Biotek, USA) at 450 nm. All samples were examined in triplicate.

### Lymphocyte proliferation assays

Two weeks after the last immunisation, the spleens were collected from the mice in each group under aseptic conditions in Hank's balanced salt mixture (Solarbio, CN), then minced using a pair of scissors and passed through a 41.6 µm mesh sieve to produce a homogeneous cell suspension. The lymphocytes were obtained using lymphocyte isolation solution (Biyuntian, CN). Then, 2×10^5^ cells/well were cultured in 96-well plates in triplicate in RPMI-1640 containing 10% FCS and either stimulated with 10 μg/ml of rTgPDI or not for 72 h at 37°C under 5% CO_2_. Cell Counting Kit-8 (Beijing Dojindo, CN) was used, and the results were expressed as the stimulation index (SI), which is the ratio of the OD_450_ of stimulated cells to the OD_450_ of unstimulated cells.

### Cytokine assays

For these assays, 1.5×10^6^ cells/well were seeded into 24-well plates in triplicate and stimulated with 10 μg of rTgPDI. Cell-free supernatants were harvested and assayed to determine IL-2 and IL-4 activities at 24 h; IL-10 activity at 72 h; and interferon-gamma (IFN-γ) activity at 96 h. The concentrations of IL-2, IL-4, IL-10 and IFN-γ were evaluated using a commercial ELISA kit (PeproTech, USA) according to the manufacturer's instructions. All assays were performed in triplicate. Cytokine concentrations were determined in reference to standard curves constructed with known amounts of mouse recombinant IL-2, IL-4, IL-10 or IFN-γ. The sensitivity limits of IL-2, IL-4, IL-10 and IFN-γ detection were 16, 16, 47 and 23 pg/ml, respectively.

### Challenge infection

According to the results regarding the doses of rTgPDI that elicited different immune responses, 30 μg of rTgPDI was selected as the dose for chronic and acute challenge infection. A total of 52 mice (half male and half female) were randomly divided into two groups (the PBS and 30 μg dose groups; 26 mice/group, 10 subjected to chronic infection and 16 to the acute assay) and challenged via intragastric administration of 1×10^4^ tachyzoites for the chronic assay or 4×10^4^ tachyzoites for acute infection on the fifteenth day after the last immunisation. The numbers of tachyzoites in the brains and livers of the mice were measured to assess the results of the chronic challenge infection assay. For survival analysis, the infected mice were monitored at 8 am, 2 pm and 8 pm daily regarding their physical appearance, such as displaying rough coat, decreases in appetite, weakness/inability to obtain feed or water and depression. When these conditions were observed, a mouse would be moved to an isolated cage for further husbandry; if obvious suffering, such as struggling or whining was observed, the mouse would be sacrificed through ether inhalation. No obvious suffering was observed in this study. To assess the protective effect of rTgPDI in the infected mice, the time to death and survival were recorded and assessed for one month after parasite challenge.

### Quantitation of *T. gondii* tachyzoites in murine brains and livers

At two weeks post-vaccination with PBS or 30 μg rTgPDI, 10 mice from each group were challenged orally with 1×10^4^ tachyzoites from the *T. gondii* RH strain. During the challenge, no suffering was observed. Four weeks later, the challenged mice were killed using an overdose of sodium pentobarbital, and the intact brains and partial livers were collected from each mouse and homogenised in 2 ml of PBS. For each brain and liver suspension, the mean number of tachyzoites was determined from four samples (25 μl each) in a hemocytometer. The tachyzoite load was estimated on the basis of the average quantity of tachyzoites per gram brain or liver tissue.

### Western blotting

The STAg was prepared as described previously [11]. The rTgPDI products expressed in *E. coli* Rosetta (DE3) and the soluble tachyzoite antigen were boiled at 95°C for 5 min, followed by centrifugation at 12,000×*g* for 10 min at room temperature. The supernatants were separated in 12% SDS-PAGE gels and then electrophoretically transferred to PVDF membranes (GE Healthcare). The membranes were blocked in 5% skim milk for 1 h at room temperature and then incubated at 4°C overnight with the anti-His primary antibody (1∶1000), rabbit anti-*T. gondii* serum (1∶200), human anti-*T. gondii* serum (1∶200) or mouse anti-*T. gondii* serum produced by immunization with rTgPDI (1∶200). The PVDF membrane was subsequently incubated with anti-mouse (rabbit or human) HRP-IgG (Beyotime, CN) for 1 h at room temperature, and chemiluminescence was detected using an ECL blot detection system (Engreen, CN).

### Statistical analysis

All data, including results of the lymphocyte proliferation assays, antibody responses, cytokine levels and the tachyzoite loads, were statistically analysised by one-way analysis of variance (ANOVA) using the Statistical Package for the Social Sciences (SPSS) for Windows, version 13.0. P values of less than 0.05 were considered statistically significant. The survival times for the vaccinated and control groups were compared using the Kaplan-Meier method.

## Results

### TgPDI is a conserved protein

**Figure 1:**
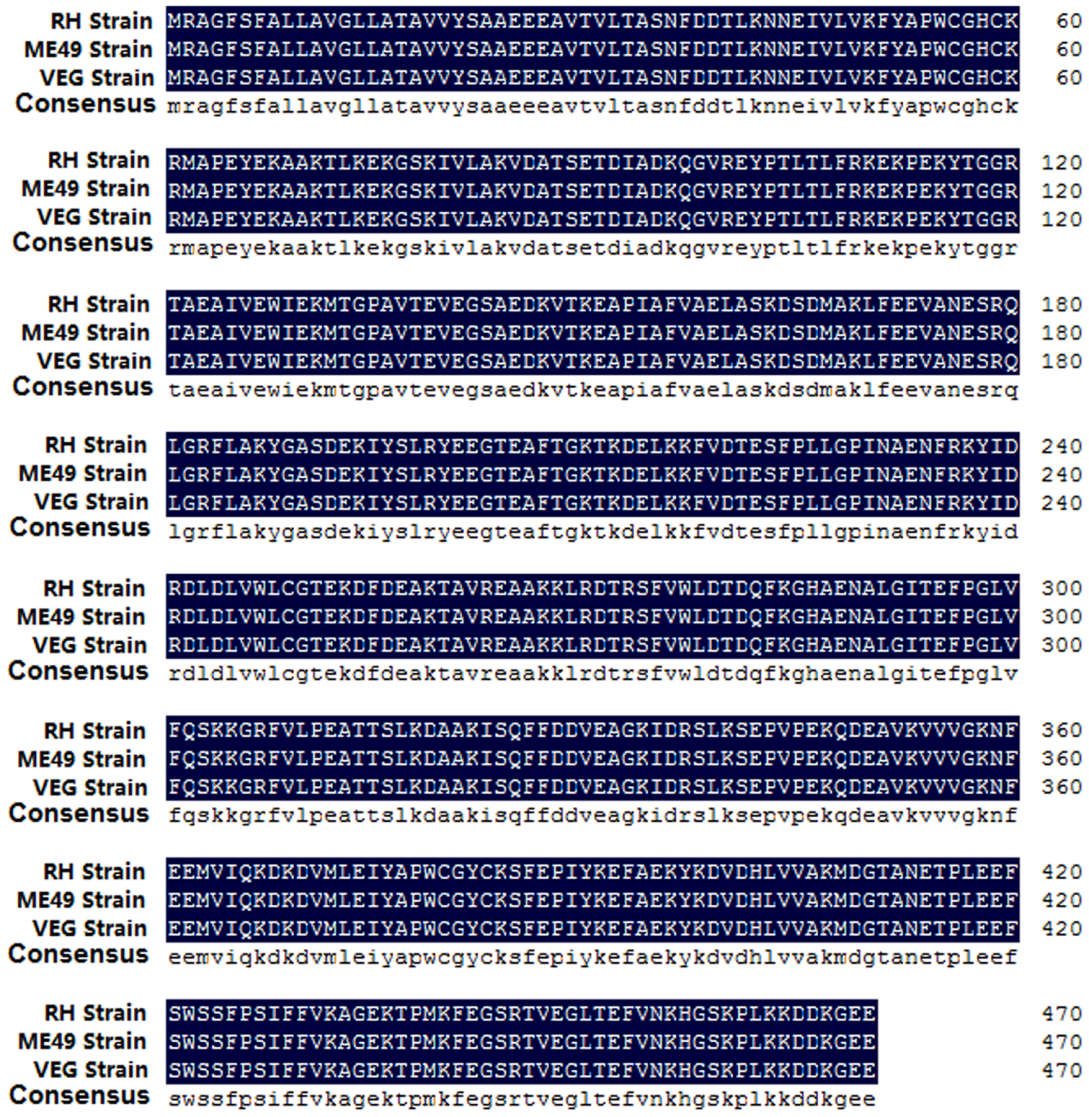
Alignment of the amino acid sequence of TgPDI. The alignment of the amino acid sequence of PDI from three strains(RH, ME49 and VEG strain)of *T. gondii* was performed by DNAMAN (Lynnon, Quebec, Canada). Homology levels are highlighted in colors. Black: 100%.

The alignment of the amino acid sequence of TgPDI shows a 100% homology among three strains which represent different genotypes of *T. gondii* (Fig. 1). The results indicate the PDI is a conserved protein in evolution of *Toxoplasma*.

### rTgPDI was expressed and purified successfully

**Figure 2:**
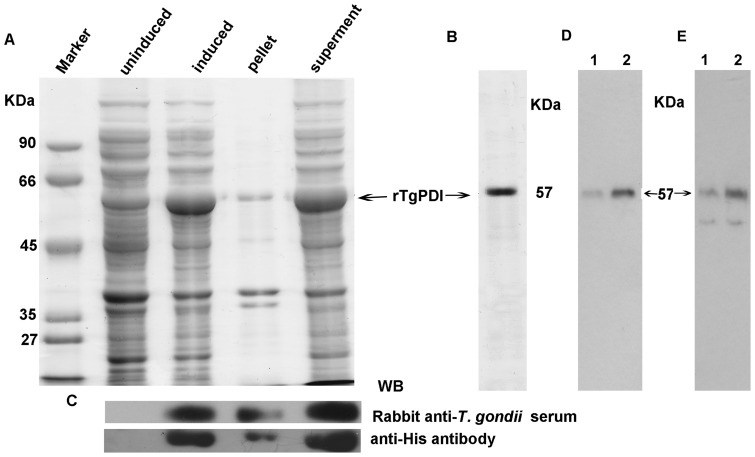
SDS-PAGE and Western blotting analyses of the rTgPDI protein. (A) The full-length ORF of TgPDI was expressed in *E. coli*, then separated on 12% SDS-PAGE gels and stained with Coomassie blue. The molecular weight of rTgPDI was approximately 57 KDa. (B) Purified rTgPDI protein was detected via 12% SDS-PAGE and staining with Coomassie blue; the purity of rTgPDI was greater than 95%. (C) Western blotting analysis of rTgPDI using a rabbit anti-*T. gondii* serum polyclonal antibody (upper) and anti-His antibody (lower); rTgPDI could be recognised by both the His antibody and anti-*T. gondii* serum. (D) Western blotting analysis of purified rTgPDI using an anti-*Toxoplasma* human serum. Lane 1 and 2 represented 2 μg and 5 μg purified rTgPDI respectively. (E) Western blotting analysis of native TgPDI using the antibodies elicited from mouse immunised with rTgPDI. Lane 1 and 2 represented 20 μg and 50 μg STAg respectively.

The cDNA sequence corresponding to the TgPDI ORF was cloned into the prokaryotic expression vector pET-30a(+) and transformed into the Rosetta (DE3) bacterial host. After induction with 0.1 mM IPTG at 25°C, the rTgPDI proteins were successfully expressed in *E. coli* and the molecular weight was approximately 57 KDa (Fig. 2A). The isolated protein was water soluble and showed 95% purity based on SDS-PAGE analysis (Fig. 2B). Western blotting analysis indicated that the rTgPDI band was able to react with the rabbit anti-*T. gondii* serum polyclonal antibody and anti-His antibody (Fig. 2C). Moreover, rTgPDI was recognised by anti-*Toxoplasma* human serum (Fig. 2D) and native TgPDI contained in the soluble tachyzoite antigen was recognized by the antibodies elicited from mouse immunised with rTgPDI (Fig. 2E).

### Humoral immune responses were induced by rTgPDI

**Figure 3:**
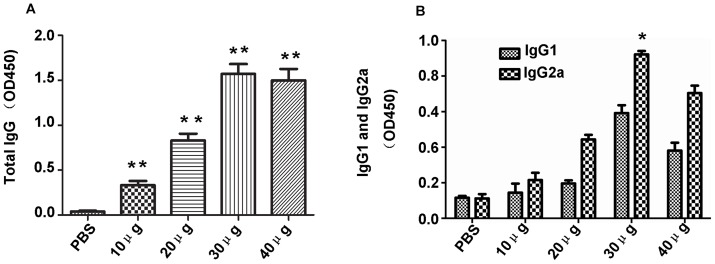
The antibody response induced by rTgPDI vaccine immunisation. Sera were collected from the BALB/c mice immunised with different doses of rTgPDI or PBS two weeks after the final inoculation. (A) Determination of the levels of specific anti-rTgPDI IgG in the sera of BALB/c mice immunised with different doses of rTgPDI or PBS under the same conditions. Results are expressed as the mean OD_450_± SD (n = 10) and are representative of three individual experiments. (B) The levels of both IgG1- and IgG2a-specific anti-rTgPDI isotype antibodies in the sera of mice immunised with different doses of rTgPDI or PBS. Results are expressed as the mean OD_450_± SD (n = 10) and are representative of three individual experiments. The results indicated that rTgPDI could induce IgG production and a Th1-type response. **P<0.01 compared with the control group.

To investigate whether humoral immune responses were induced in mice immunised with different doses of the rTgPDI protein, collected sera were tested by ELISA. A total IgG antibody response was observed in all of the groups, except the PBS-treated control group. There was a significant antibody response detected in immunised group compared to the control (*P*<0.01) (Fig. 3A). The results also showed that 30 μg of rTgPDI elicited the maximum IgG antibody values among the tested doses (*P*<0.05). However, there was no significant difference in IgG responses observed between the groups immunised with 30 or 40 μg of rTgPDI (*P*>0.05).

The levels of the IgG1 and IgG2a humoral isotype responses in immunised BALB/c mice were evaluated by ELISA. A mixed IgG1/IgG2a response, involving predominant IgG2a production was detected in the sera of mice immunised with rTgPDI (Fig. 3B). These results indicated a shift toward a Th1-type response. Moreover, immunisation of mice with 30 μg of rTgPDI elicited higher levels of IgG2a compared to the control and other dosage groups (*P*<0.05).

**Figure 4:**
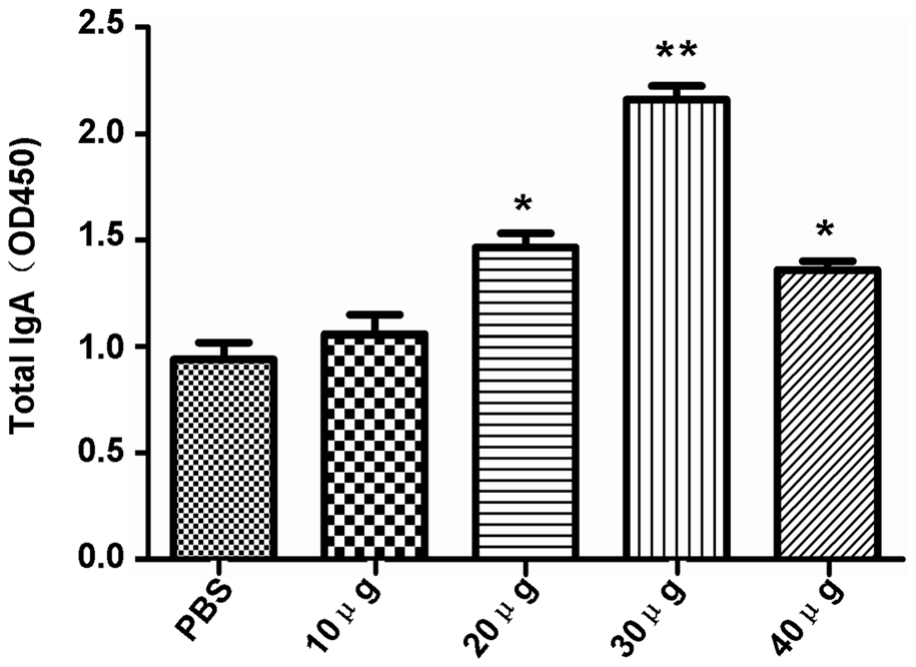
Specific anti-rTgPDI IgA antibody responses in immunised mice. Sera were collected from BALB/c mice immunised with different doses of rTgPDI or PBS on day 35 and analysed by ELISA. Results are expressed as the mean of the OD_450_± SD (n = 10) and are representative of three individual experiments. Administration of 30 μg of rTgPDI stimulated significantly higher IgA production compared with the other rTgPDI doses and PBS. *P<0.05 and **P<0.01 compared with control group.

Total IgA antibody levels were also measured. There was a significant antibody response in the 20 μg and 40 μg dose groups compared to the control group (*P*<0.05) (Fig. 4). Similarly, 30 μg of rTgPDI elicited the highest IgA antibody levels compared with the control group (*P*<0.01). These findings revealed that a mucosal immune response was elicited by intranasal rTgPDI immunisation.

### rTgPDI stimulated lymphocyte proliferation

**Figure 5:**
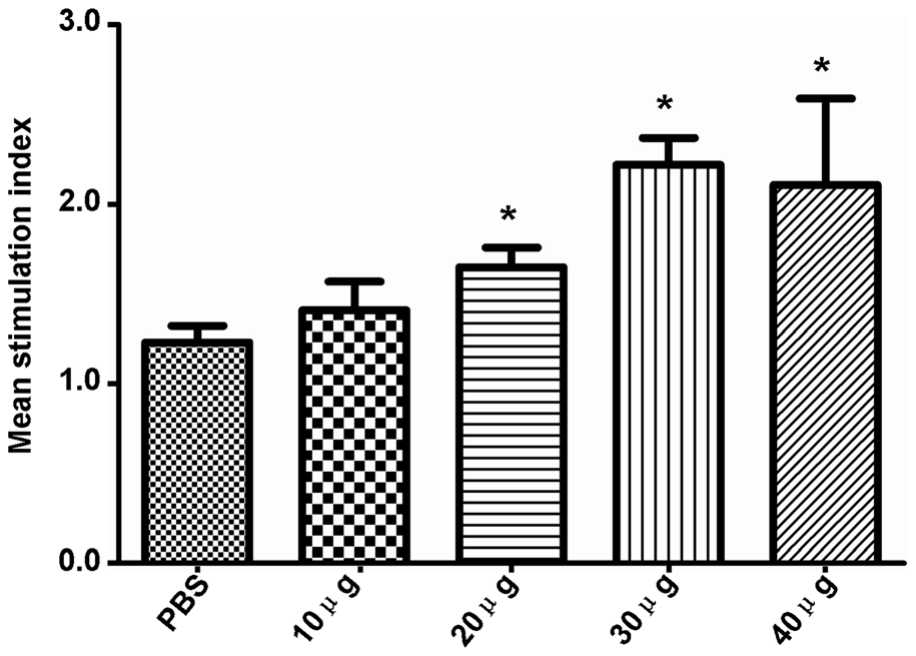
Splenocyte proliferation responses in mice immunised with rTgPDI or PBS. Spleen lymphocytes were collected from the BALB/c mice immunised with different doses of rTgPDI or PBS two weeks after the last immunisation using 10 μg of rTgPDI as a stimulus. Results are expressed as mean stimulation index (SI) ± SD (n = 10) and are representative of three individual experiments. The data showed that 30 μg of rTgPDI could evoke an obvious splenocyte proliferation response compared with the other rTgPDI doses and PBS. *P<0.05 compared with the control group.

Splenocytes from mice immunised with different doses of rTgPDI or PBS were prepared to assess cell-mediated immune responses. The lymphocyte proliferative response assay was performed using 10 μg rTgPDI as a stimulus. As shown in Fig. 5, the specific proliferative response was significantly stronger in mice immunised with 20, 30 or 40 μg of rTgPDI (*P*<0.05) than in mice immunised with PBS. However, there was no significant difference between the 10 μg rTgPDI and PBS groups (*P*>0.05).

### Th1-type cellular immune responses were strongly elicited by rTgPDI

**Figure 6:**
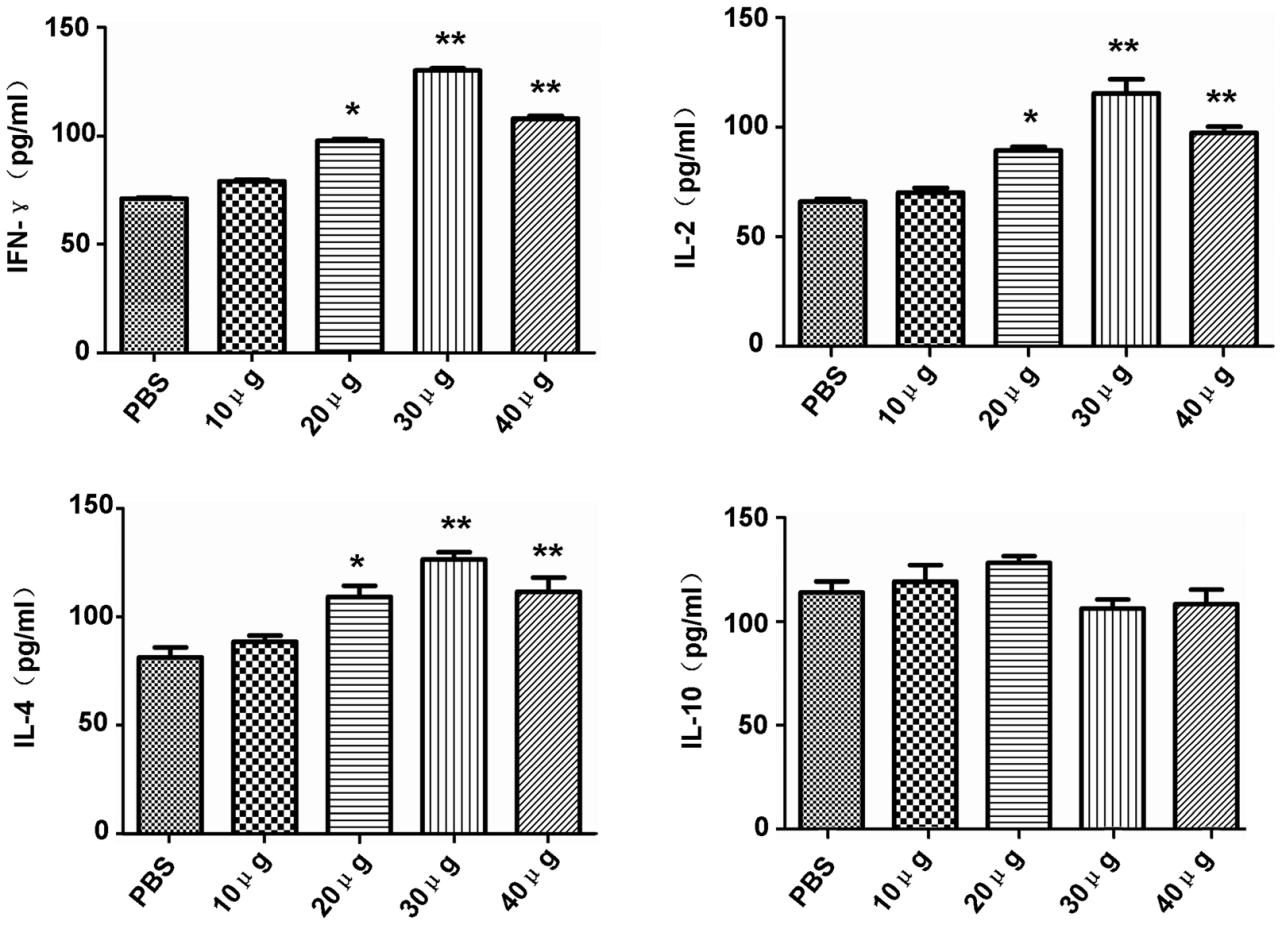
Cytokine production in splenocytes from immunised mice. The culture supernatants were examined by ELISA for cytokine production. The IL-2 and IL-4 levels were determined after 24 h of culturing; IL-10 activity after 72 h; and IFN-γ levels after 96 h. The results showed that rTgPDI (especially the 30 μg dosage) increased the levels of IFN-γ, IL-2 and IL-4, but not IL-10 compared with the PBS control. *P<0.05 and **P<0.01 compared with the control group.

The cell-mediated immunity induced in the immunised mice was evaluated by measuring the levels of cytokines (IFN-γ, IL-2, IL-4 and IL-10) released in the supernatants of cultures of rTgPDI-stimulated spleen cells. The levels of IFN-γ, IL-2 and IL-4 secreted from the spleen cells of all of the rTgPDI-immunised mice, except those in the 10 μg dose group, were increased compared with the PBS group, and 30 μg of rTgPDI provoked cell-mediated immunity more efficiently compared to the other dosages (*P*<0.01). Nevertheless, the level of IL-10 among the different groups showed no significant changes (*P*>0.05) (Fig. 6). Generally, IFN-γ and IL-2 favour Th1-type immune responses, whereas IL-4 and IL-10 are correlated with a Th2-type response. Therefore, these data indicated that the cellular immune response induced by rTgPDI was oriented toward a Th1 profile in the immunised mice.

### rTgPDI reduced the burden of *T. gondii* tachyzoites

**Figure 7:**
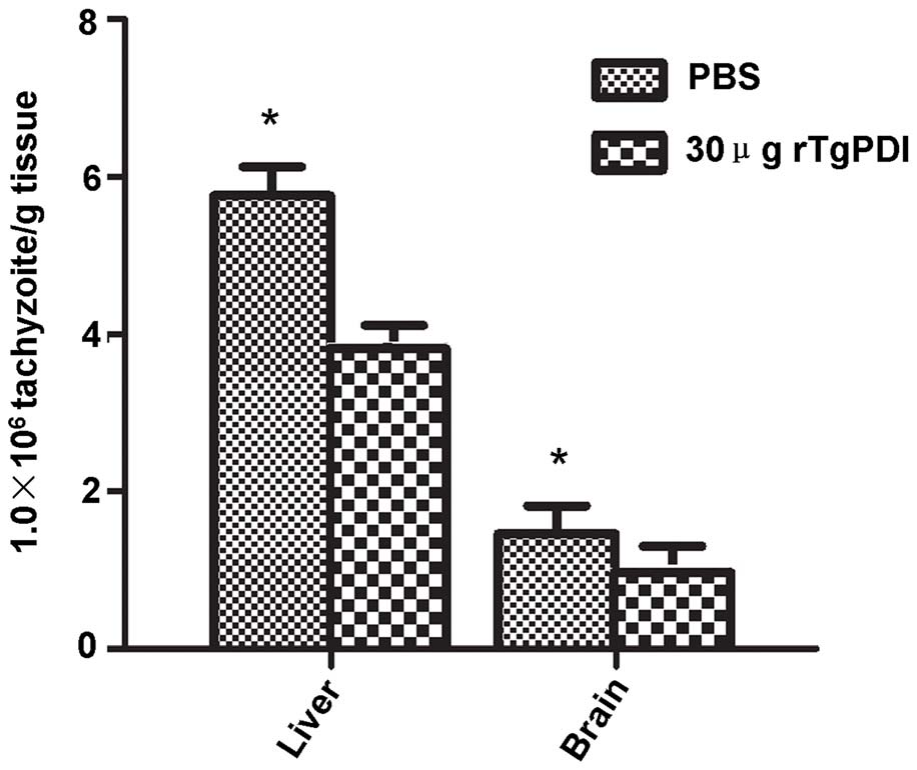
The burden of tachyzoites in immunised mice. The brains and livers of immunised mice and control mice were collected, and the tachyzoite burdens were quantified via microscopy. Results are expressed as the mean ± SD (n = 10) and represent numbers of tachyzoites per gram of tissue. The data showed that rTgPDI could reduce the tachyzoite burdens in the brain and liver compared with the control group. *P<0.05 compared with the control group.

Lower brain and liver tachyzoite burdens (*P*<0.05) were observed in mice immunised with 30 μg of rTgPDI protein compared with the control group (Fig. 7). These findings suggested that the immunity elicited by rTgPDI was able to efficiently prevent the invasion or proliferation of tachyzoites in host tissues.

### rTgPDI prolonged the survival of lethally challenged BALB/c mice

**Figure 8:**
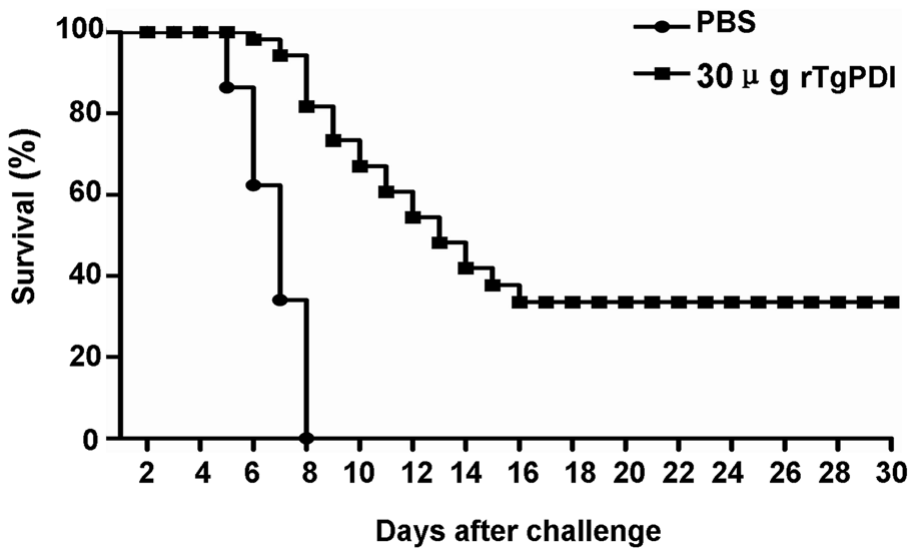
Survival of the immunised mice after *T. gondii* challenge. Two groups of mice (16 mice per group) were immunised with 30 μg of rTgPDI or PBS through intranasal administration at 0, 14 and 21 days, and on the 15th day after the last immunisation, the mice were orally challenged with 4×10^4^ *T. gondii* tachyzoites from the RH strain. The survival of the mice was monitored daily for 30 days after challenge. The results showed that treatment with 30 μg of rTgPDI prolonged the survival of immunised BALB/c mice compared to the controls.

To evaluate whether the rTgPDI protein could induce a protective effect against a lethal *T. gondii* infection, mice were orally infected with 4×10^4^ tachyzoites on the fifteenth day after the last immunisation. The resultant survival curves are shown in Fig. 8. The survival time for mice immunised with 30 μg of rTgPDI was significantly longer (*P*<0.05) compared to the control group. All mice in the control group died from 5 to 8 days after challenge, whereas the immunised mice showed protection from infection. Immunisation of the mice with 30 μg of rTgPDI significantly increased their survival rate on the 30th day after challenge by approximately 31% compared with control group (*P*<0.05). Interestingly, one of survived mice was female and others were male.

## Discussion

Recently, proteins responsible for parasite moving junction development, gliding motility, and adhesion to host cells and proteins that serve as essential determinants of parasite virulence and invasion have been investigated as vaccine candidates for combating *T. gondii* infection, including surface proteins, microneme proteins, rhoptry proteins [26]–[30] and dense granule proteins [31], [32]. Most of previous studies focused on using DNA vaccines or DNA-cocktail vaccines, with few protein vaccines being tested. It is well known that protein vaccines elicit strong humoral responses by inducing antigen-specific antibodies. DNA vaccines though induce cellular immune responses [33] and priming of antigen-specific memory B-cells [34], are still suffering from low efficacy in immunogenicity. Therefore, protein vaccines should be emphasised in attempting to develop a vaccine against toxoplasmosis.

It has been revealed that the majority of *T. gondii* strains comprises three distinct clonal lineages [35], [36] with a genetic difference of 1% or less [37]. These genotypes have highly different phenotypes in the laboratory mice, with type I strains being acutely virulent, type II strains intermediate virulence, and type III strains essentially avirulent [38]. PDI is highly expressed in *T. gondii* and acts as chaperones to aid protein folding by forming a bridge between two cysteines [39], as well as modulates the tachyzoite-host cell interaction [22], [23]. In addition, PDI is a conserved protein in evolution of *Toxoplasma* as the alignment of the amino acid sequence of PDI revealed a 100% homology among three strains of *T. gondii* (Fig. 1). Theoretically, the protection elicited by TgPDI immunization can protect against all three strains.

In present study, the plasmid pET-30a(+)-TgPDI was constructed, and the rTgPDI protein, which could be recognised by anti-*T. gondii* serum, was expressed in *E. coli* and purified using Ni^2+^-NTA agarose. It is well established that IFN-γ, IL-2 and IgG2a expression is correlated with a Th1-type response, while IL-4, IL-10 and IgG1 favour a Th2-type immune response [40], [41]. Most importantly, IFN-γ plays a critical role in protective immunity against *T. gondii* [42]. Our dose-dependence analyses indicated that 30 μg of rTgPDI evoked a significant Th1-type immune response, as demonstrated by high levels of IFN-γ, IL-2 and IgG2a (*P*<0.01), while no obvious changes in IL-10 of Th2-type immune response. In addition to cellular immune responses, both humoral immunity, associated with increased total IgG antibody levels, and mucosal immune responses, resulting in IgA production, are important in controlling *T. gondii* infection [43], [44]. Our results showed that the administration of 30 μg of rTgPDI resulted in higher levels of IgG and IgA in sera compared with the other treatments. The findings described above are consistent with previous studies examining subunit vaccines for toxoplasmosis, which showed a mixed Th1/Th2 but predominantly Th1-type immune response by immunisation with recombinant proteins [45].

For 30 μg of rTgPDI elicited the strongest cellular and humoral immune responses in this study, this dosage was used to evaluate the protective effect of rTgPDI against *T. gondii* challenge. Our data suggested that 30 μg of rTgPDI reduced tachyzoite loads in mouse brain and liver by 42% and 37%, respectively. Considerable levels of IgA antibodies were produced in the 30 μg of rTgPDI group, which is correlated with mucosal immunity and protection against pathogen infestation of local mucous membranes, such as the intestinal mucosa. Therefore, we infer that the invasion or proliferation of tachyzoites in immunised host might be suppressed by the antibodies, cytokines and immune cells elicited by rTgPDI treatment, potentially through affecting the tachyzoite-host cell interaction [22], [23] and proper folding of pathogenic proteins [39]. Furthermore, prolonged survival was observed in immunised BALB/c mice, with approximately 31% of the immunised mice remaining alive at the end of analysis period. These results demonstrated that rTgPDI treatment provided partial, but effective protection against both chronic and acute *T. gondii* infection.

As an indispensable part of vaccine, adjuvant plays a critical role in enhancing immunogenicity of weaker antigen and activating an appropriate immune response safely without multiple dose regimens. Monophosphoryl lipid A (MPL), oligodeoxynucleotides containing unmethylated CpG motifs and cytokines such as interleukin-12 (IL-12), acting as Th1-promoting immunopotentiators, exhibit desirable adjuvant activities that are essential for immunisation against *T. gondii* [26], [46]–[48]. Therefore, to assess the degree of rTgPDI conservation, further studies should be performed involving different genotypes of *T. gondii* isolates, in conjunction with other antigens or appreciate adjuvants.

In this study, we used rTgPDI as antigen for a protein vaccine. The rTgPDI was expressed in soluble manner in *E. coli* ensuring proper processing of necessary functional groups of the recombinant protein. rTgPDI can be recognized by anti-*Toxoplasma* rabbit or human serum. Native TgPDI in the soluble tachyzoite antigen was recognized by antibodies elicited from mouse immunised with rTgPDI. These results show that TgPDI has antigenic property. Importantly, inoculation route and challenge manner in this study were intranasal administration and oral infection rather than traditional intramuscular injection and intraperitoneal infection [49]–[51]. Intranasal immunisation can induce mucosal immune responses which can inactivate the pathogen and contribute to pathogen elimination [52]. Oral challenge is more reasonable because *T. gondii* is an orally acquired apicomplexan protozoan parasite. In our study, about 31% infected mice immunised with rTgPDI were kept alive till the end of study period. Our results showed clear protective effects by rTgPDI in survival time and survival rate compared with other recombinant proteins such as recombinant *Toxoplasma gondii* actin depolymerizing factor and recombinant nucleoside triphosphate hydrolase-II. In their studies, all immunised mice all died within 9 or 14 days respectively [45], [51].

BALB/c mice are commonly used for vaccine against *Toxoplasma* infection. In general, females tend to exhibit better humoral immunity than males. Males manifest greater levels of cellular immunity [53]. Female mice were more susceptible to acute infection, while surviving female mice with chronic infections harbored more cysts in their brains than did surviving males [54]. Taking these differences into account, both male and female mice were used in our study. Data from both male and female were averaged for eliminating gender immune differences. In acute infection assays, five mice (about 31%) survived, one was female and others were male. These results are in accordance with the observation that female mice are more susceptible and more likely to die from *T. gondii* infection than males [54], [55]. A shortback in this study is that the kinetics and strength of the immune response in male and female rTgPDI-immunised BALB/c mice have not been observed. Therefore, the kinetics and strength of the immune response assays should be explored in future study using rTgPDI combined with appropriate adjuvants based on the present data.

In summary, the present study evaluated the immunogenicity and protective potency of rTgPDI as a protein vaccine. This protein was able to elicit significant humoral, mucosal and cellular immune responses of a Th1 type, which significantly reduced the tachyzoite loads in different tissues and increased the survival of BALB/c mice challenged with tachyzoites from the lethal *T. gondii* RH strain. This exploration demonstrated that PDI might represent a promising anti-toxoplasmosis vaccine candidate.
